# Serum Non-Esterified Fatty Acids, Carotid Artery Intima-Media Thickness and Flow-Mediated Dilation in Older Adults: The Cardiovascular Health Study (CHS)

**DOI:** 10.3390/nu13093052

**Published:** 2021-08-31

**Authors:** Neil K. Huang, Petra Bůžková, Nirupa R. Matthan, Luc Djoussé, Jorge R. Kizer, Kenneth J. Mukamal, Joseph F. Polak, Alice H. Lichtenstein

**Affiliations:** 1 Cardiovascular Nutrition Laboratory, Jean Mayer USDA Human Nutrition Research Center on Aging, Tufts University, Boston, MA 02111, USA; neil.huang@tufts.edu (N.K.H.); nirupa.matthan@tufts.edu (N.R.M.); 2 Department of Biostatistics, University of Washington, Seattle, WA 98115, USA; buzkova@uw.edu; 3 Division of Aging, Brigham and Women’s Hospital, Harvard Medical School, Boston, MA 02115, USA; ldjousse@rics.bwh.harvard.edu; 4 Cardiology Section, San Francisco Veterans Affairs Health Care System, San Francisco, CA 94121, USA; jorge.kizer@ucsf.edu; 5 Department of Medicine, Epidemiology and Biostatistics, University of California San Francisco, San Francisco, CA 94158, USA; 6 Beth Israel Deaconess Medical Center, Division of General Medicine, Boston, MA 02215, USA; kmukamal@bidmc.harvard.edu; 7 Department of Radiology, Tufts Medical Center, Boston, MA 02111, USA; jpolak@tuftsmedicalcenter.org

**Keywords:** serum non-esterified fatty acid, conjugated linoleic acid, palmitoleic acid, carotid intima-media thickness, flow-mediated dilation, Cardiovascular Health Study

## Abstract

**Backgrounds and aims:** Elevated common carotid artery intima-media thickness (carotid IMT) and diminished flow-mediated dilation (FMD) are early subclinical indicators of atherosclerosis. Serum total non-esterified fatty acid (NEFA) concentrations have been positively associated with subclinical atherosclerosis. The relations between individual NEFA, carotid IMT and FMD have as yet to be assessed. **Methods:** We investigated the associations between fasting serum individual NEFA, carotid IMT and FMD among Cardiovascular Health Study (CHS) participants with (*n* = 255 for carotid IMT, 301 for FMD) or without (*n* = 1314 for carotid IMT, 1462 for FMD) known atherosclerotic cardiovascular disease (ASCVD). Using archived samples (fasting) collected from 1996–1997 (baseline), 35 individual NEFAs were measured using gas chromatography. Carotid IMT and estimated plaque thickness (mean of maximum internal carotid IMT) were determined in 1998–1999. FMD was measured in 1997–1998. Linear regression adjusted by the Holm-Bonferroni method was used to assess relations between individual NEFA, carotid IMT and FMD. **Results:** In multivariable adjusted linear regression models per SD increment, the non-esterified *trans* fatty acid conjugated linoleic acid (*trans*-18:2 CLA) was positively associated with carotid IMT [β (95% CI): 44.8 (19.2, 70.4), *p* = 0.025] among participants with, but not without, ASCVD [2.16 (−6.74, 11.5), *p* = 1.000]. Non-esterified *cis*-palmitoleic acid (16:1n-7*c*) was positively associated with FMD [19.7 (8.34, 31.0), *p* = 0.024] among participants without, but not with ASCVD. No significant associations between NEFAs and estimated plaque thickness were observed. **Conclusions:** In older adults, serum non-esterified CLA and palmitoleic acid were positively associated with carotid IMT and FMD, respectively, suggesting potential modifiable biomarkers for arteriopathy.

## 1. Introduction

Cardiovascular disease (CVD) is the most common and leading cause of morbidity and mortality in the US [1]. Among individuals over the age of 65 years, subclinical atherosclerosis is estimated to affect over one-third of both women and men [2,3].

Elevated carotid intima-media thickness (carotid IMT) is an indicator of nascent plaque build-up [4]. Inflammation and disorders of lipoprotein metabolism are positively associated with carotid IMT [5]. Flow-mediated dilation (FMD) is a noninvasive measure of endothelial function and considered a reflection of endothelial nitric oxide synthase (eNOS) derived nitric oxide production. Diminished FMD is positively associated with impaired eNOS activity and CVD risk [6,7,8]. Both FMD and carotid IMT have been associated with risk of clinical cardiovascular disease in the Cardiovascular Health Study [6,9].

Circulating non-esterified fatty acids (NEFA) originate from adipose tissue during fasting and triglyceride hydrolysis during the postprandial state. They vary chain length and degree of saturation. Circulating total NEFA concentrations have been associated with local and systemic inflammation [10], induction of oxidative stress [10], endothelial dysfunction [11] and foam cell formation [11,12,13]. The relation of individual NEFA species with measures of subclinical atherosclerosis has yet to be determined.

The aims of this study were to assess the relations of fasting serum individual NEFAs with carotid IMT and FMD, and as a secondary outcome, estimated plaque thickness, in the CHS cohort. We hypothesized that fasting serum individual non-esterified unsaturated FA would be negatively associated, whereas saturated FAs and *trans* FAs would be positively associated, with carotid IMT and estimated plaque thickness, and the opposite associations with FMD.

## 2. Materials and Methods

### 2.1. Study Design and Population

The CHS is a population-based, longitudinal study of risk factors for cardiovascular disease in U.S. adults aged 65 years and older, and the information regarding sample size calculation, study design and rationale of this cohort was published previously [14]. During 1989–1990, a total of 5201 Medicare-eligible residents were recruited in 4 U.S. clinical sites (Allegheny County, PA; Forsyth County, NC; Sacramento County, CA; Washington County, MD). In 1992–1993, similar methods were used to recruit 687 Black participants from the same field centers with the exception of Washington County, MD. Participants attended clinic exams at baseline and annually through 1999. Of the 4413 participants who attended the 1996–1997 visit, NEFA profiles were determined in 2145 participants who had an unthawed fasting serum specimen (fasting at least 8 hours) and were not using hypoglycemic medications. After excluding specimens that were hemolyzed (*n* = 5), NEFA profiles were determined for 2140 participants. Among those participants, 1569 had a carotid IMT measurement at their 1998–1999 visit, and 1763 underwent FMD measurement at their 1997–1998 visit. For each outcome, participants were divided on the basis of whether they were diagnosed with or without atherosclerotic cardiovascular diseases (ASCVD) based on the available records, including congestive heart failure, myocardial infarction, stroke and peripheral artery disease. For carotid IMT, we included 1314 participants without and 255 participants with a ASCVD diagnosis. For estimated plaque thickness, we included 1314 participants without a ASCVD diagnosis. For FMD, we included 1462 without and 301 participants with a ASCVD group diagnosis.

### 2.2. Non-Esterified Fatty Acid (µmol/L) Determinations

All fasting samples used for NEFA analysis were collected in 1996–1997 and analyzed in 2017. These samples were stored at −80 °C and not thawed prior to the NEFA analyses. Antioxidants or triglyceride lipolysis inhibitors were not added at the time of sample collection. No evidence of oxidation or degradation products were observed based on the gas chromatograms. Lipids were extracted from serum using a modified Folch method [15,16,17] after addition of an internal standard (heptadecanoic acid). The serum NEFA fraction was isolated using solid-phase chromatography (aminopropyl columns), saponified, methylated, and the resulting fatty acid methyl esters were quantified using an Autosystem XL gas chromatograph (Perkin Elmer, Boston, MA, USA) equipped with a 100 m × 0.25 mm capillary column (HP INNOWQAX, Agilent Technologies, Wilmington, DE, USA) [18]. Thirty-five individual fatty acids were identified by comparison with authenticated standards (NuCheck Prep, MN, USA). A pooled human serum sample as a quality control was included at the beginning and the middle of each batch of samples. The intra- and inter-assay coefficients of variation were 0.5 to 4.3% for fatty acids present at >25 µmol/L, 1.8 to 7.1% for fatty acids present at proportions between 5–25 µmol/L, and 2.8 to 11.1% for fatty acids present at proportions <5 µmol/L. The NEFA analysis was conducted at the Jean Mayer USDA Human Nutrition Research Center on Aging at Tufts University, Boston, MA, USA.

### 2.3. Carotid Intima-Media Thickness and Flow-Mediated Dilation

Carotid IMT was measured at the 1998–1999 visit using sonographic equipment (Toshiba SSA-270A, Toshiba American Medical Systems, Tustin, CA, USA). The same equipment was used in all field centers. It was calibrated periodically using tissue-equivalent phantom, and all carotid IMT data were analyzed at the Ultrasound Reading Center. The protocol was described previously [19]. In brief, determined were three longitudinal views centered on the largest lesion in the right and left internal carotid arteries and a single lateral view of the distal 10 mm of the common carotid artery. The internal carotid artery was defined as the carotid bulb and the initial 10 mm of vessel distal to the tip of the flow divider and measures began with dilation of the carotid bulb with loss of the parallel configuration of the near and far walls of the common carotid artery. As in previous studies, carotid IMT was calculated as the sum of the maximum common carotid IMT at the far wall divided by its standard deviation (SD) and the maximum internal carotid IMT at the far wall divided by its SD [19]. Secondarily, we examined the mean of the maximum internal carotid IMT as a measure of estimated plaque thickness (cutoff: 1.5 mm) in participants without ASCVD [20].

Brachial FMD was measured in 1997–1998 using ultrasound images instruments, which included a 7.5 MHz linear array transducer from Toshiba SSA 270A (Toshiba American Medical Systems, Tustin, CA, USA), a 9 MHz annular array transducer from Biosound Phase II, 7.5 and 10 MHz linear array transducers from Acuson Aspen and a 13 MHz linear array transducer from Acuson Sequoia. The same equipment was used in all field centers, and the sonographers underwent centralized training in brachial FMD measurement at the School of Medicine, Wake Forest University and were certified after the training was completed. As described previously [21], following an 8 h fast to avoid postprandial attenuation of FMD [22], a standard sphygmomanometer cuff was placed on the right forearm 2 inches below antecubital fossa. Participants reclined flat on the table in a temperature-controlled room (22 °C) for 15 min before measurements. Baseline brachial artery images were recorded for 1 min with the deflated cuff, and then 4 min post-inflation of the cuff to 50 mmHg above the participant’s resting systolic pressure. The cuff was then deflated, and the images were recorded for additional 2 min. All acquired FMD images were analyzed at the Cardiology Image Processing Laboratory at Wake Forest University using a validated semiautomated system, which generated the baseline and maximum diameters of the brachial artery. With semiautomated analysis, the correlations for intra-reader, inter-reader, and intra-subject ranged from 0.67 to 0.84 for the repeated measures of percent change in diameter [22]. %FMD was calculated as [(Maximum diameter − baseline diameter)/baseline diameter] × 100% [21].

### 2.4. Other Covariates

All other participant characteristics for the current study were collected at the 1996–1997 visit, which serves as study baseline. The following covariates were adjusted for the statistical analyses: smoking status (never, former, and current), alcohol intake (none, 1–7 drinks/week, >7 drinks/week), and health status were assessed by questionnaire [14]. Serum albumin was measured using a standardized method and included as a covariate. Weight, height, and waist circumference were measured using standard methodology. C-reactive protein was measured using enzyme-linked immunosorbent assay [23]. Physical activity was assessed using Minnesota Leisure-Time Activities questionnaire and quantified as metabolic equivalents per week [24]. Renal function was assessed based on cystatin C for estimate glomerular filtration rate (eGFR_cys_, mL/min/1.73 m^2^). Diabetes mellitus was defined as fasting glucose ≥ 7 mmol/L (126 mg/dL), non-fasting glucose ≥ 11.1 mmol/L (200 mg/dL) or use of oral hypoglycemic medications or insulin. Hypertension was defined as systolic blood pressure ≥ 140 mm Hg, diastolic blood pressure ≥ 90 mm Hg, or treatment with blood pressure lowering medications plus reported physician diagnosis of hypertension.

### 2.5. Statistical Analysis

To characterize the study population at the analysis baseline, we calculated means and standard deviations for continuous measures and percentages for binary and categorical variables. Individual NEFAs were expressed as absolute serum concentration (µmol/L) and were scaled to per SD in modeling associations with carotid IMT and % increase in FMD. Linear regression was used to estimate the associations between individual NEFAs, carotid IMT and FMD, and poisson regression was used to estimate the relative risks between individual NEFAs and estimated plaque thickness. The Holm-Bonferroni procedure was used to account for multiple comparisons (FWER < 0.05).

Multivariable regression analysis was adjusted as follows: age, sex, race, field center, education, smoking, body mass index, physical activity, alcohol intake, eGFR, serum albumin, hypertension, diabetes, use of anti-hypertensive, statin, and other lipid-lowering drugs.

All analyses were conducted using R software (version 3.6.3; Vienna, Austria), and statistical significance was defined as adjusted *p* < 0.05 (*p* < 0.00014286).

## 3. Results

### 3.1. Characteristics of Study Participants

Overall, the distributions of the participants’ characteristics at study baseline were similar among subgroups. Over two-thirds were female, and one-seventh were African American. Participants with clinical ASCVD were more likely to be former smokers and have diabetes (Table 1).

In terms of NEFA range, the distributions of NEFAs in each subgroup were similar (Supplemental Table S1). The most abundant fasting serum individual NEFAs were oleic acid (29.8–30.4%; percentage of total FA), palmitic acid (25.1–25.3%), linoleic acid (15.8–16.0%), stearic acid (12.2–12.7%) and palmitoleic acid (3.21–3.32%), contributing to 86.7–87.0% of total fasting serum NEFAs in the baseline sample.

### 3.2. Association of Serum NEFAs, Carotid Intima-Media Thickness and Estimated Plaque Thickness

Concentrations for the 35 individual NEFAs on the basis of carotid IMT participants with or without ASCVD are presented in Supplemental Table S2, and key findings are presented in Table 2. The non-esterified *trans* fatty acid conjugated linoleic acid (*trans* 18:2 CLA) was positively associated with carotid IMT [β (95% confidence interval): 44.8 (19.2, 70.4), adjusted *p* = 0.025]. No significant associations were observed among participants without preexisting ASCVD. Non-significant trends were observed for two additional *trans* FAs; non-esterified 18:1n-10–12*t* [the sum of 10 to12 *trans* isomers of 18:1; 39.4 (14.0, 64.7), adjusted *p* = 0.085] and elaidic acid [18:1n-9*t*; 38.6 (14.1, 63.1), adjusted *p* = 0.077]. No significant associations between individual NEFAs and estimated plaque thickness were observed (Supplemental Table S3).

### 3.3. Association of Serum Non-Esterified FAs and Flow-Mediated Dilation

Concentrations for the 35 individual NEFAs on the basis of FMD are presented in Supplemental Table S4 and key findings in Table 2. In the multivariable fully adjusted linear regression model, non-esterified palmitoleic acid (16:1n-7*c*) was positively associated with FMD per each SD increment in participants without preexisting ASCVD [19.7 (8.34, 31.0), adjusted *p* = 0.024]; while no significant association was observed in participants with preexisting ASCVD.

## 4. Discussion

This is the first report to assess the associations between individual NEFAs and two measures of subclinical atherosclerosis in a community-based prospective study among older adults. In individuals with ASCVD, the non-esterified *trans* FA18:2CLA was positively associated with carotid IMT, and there was a trend towards significance for two other *trans* species. In individuals with no preexisting ASCVD, the non-esterified palmitoleic acid (16:1n-7*c*) was positively associated with FMD. Other fasting serum individual NEFAs were not significantly related to carotid IMT and FMD, nor a secondary outcome, estimated plaque thickness.

The association between the fasting non-esterified 18:2 CLA and carotid IMT in individuals with preexisting ASCVD suggests a potential link between *trans* FA and arteriopathy, consistent with the trend toward an association with two other *trans* FAs, non-esterified 18:1n-10–12 *trans* isomers and elaidic acid (18:1n-9*t*). The association of 18:2 CLA with higher carotid IMT was limited to in participants with advanced disease. Dietary sources of *trans* FA include partially-hydrogenated fat and ruminant (meat and dairy) fat. Particularly during the time period of sample collection, *trans* FAs were primarily derived from the former. Total *trans* FAs have been consistently associated with dyslipidemia and elevated CVD risk [25]. However, differential effects have been observed among individual *trans* FA, particularly CLA, and carotid IMT in different cohorts [26,27,28,29,30,31,32]. Sources of these inconsistencies may be due to the different blood fractions in which FA profiles were measured [33], relative amount of different *trans* FA isomers [26,27], and analytical precision to distinguish among the *trans* FA isomers. Given that circulating NEFAs, removed from blood very rapidly and being oxidized to meet energy requirement, originate from adipose tissue during fasting status, this may reflect the sources from adipose tissues and, possibly, habitual diet. Of note, since the time the serum samples were collected, there has been a dramatic decline in the amount of partially-hydrogenated fat in the U.S. food supply [34], promoted by the Food and Drug Administration mandating the inclusion of *trans* FAs in the Nutrient Facts labels and removal from the Generally Recognized as Safe list.

The positive, favorable, association of non-esterified palmitoleic acid (16:1n-7*c*) with %FMD in individuals with no preexisting ASCVD is not without precedent. It has been postulated that palmitoleic acid can act as a lipokine, and as such reduce hepatic de novo lipogenesis and improve insulin sensitivity, inflammation, and plasma lipid profiles [35]. In CHS cohort, plasma phospholipid palmitoleic acid has been inversely associated with low-density lipoprotein cholesterol, fibrinogen concentrations and total cholesterol: high-density lipoprotein cholesterol ratio [36]. In another cohort, subcutaneous adipose tissue palmitoleic acid content was associated with lower non-fatal acute myocardial infarction risk [37]. Palmitoleic acid was the 5th highest NEFA in fasting CHS serum samples, yet amounts in the U.S. diet are low, with sources for the most part limited to macadamia nuts and seabuckthorn oil. These data suggest the majority of palmitoleic acid in circulation comes from hepatic de novo lipogenesis. In the CHS, non-esterified palmitoleic acid may be an alternative biomarker for arterial injury.

In this study, the objective of this study was to assess the relations between individual NEFAs and carotid IMT, FMD, and plaque thickness in older adults. A previous study revealed that coronary artery calcium score might improve prediction of CVD and coronary heart disease better than carotid IMT [38], but from the prediction standpoint, there were similar for stroke and transient ischemic attack, which were categorized as atherosclerotic cardiovascular disease in this study. Therefore, future studies are warranted to assess the relation between individual NEFAs and coronary artery calcium score.

A strength of this study is that the serum samples, carotid IMT and FMD data were collected in well-established research centers that also compiled extensive data on demographics, cardiometabolic risk factors and lifestyle. The analytical coefficients of variation for individual NEFAs were low, particularly for those present at low concentrations. A limitation is that only older adults who were white or black were included in the CHS cohort, which reduces the generalizability of the findings. There was possible that asymptomatic carotid artery stenosis existed, although previous data indicates the incidence is likely rare [39]. The focus of this investigation was to assess the relation between individual NEFAs and two markers for arteriopathy, and additional study is warranted to confirm the findings for arteriopathy. Although carotid artery revascularization, an alternative event outcome of subclinical atherosclerosis has been linked to carotid artery stenosis, time to event evaluation was not available [40]. We were unable to account for the differences observed between those with and without established ASCVD, although the findings for carotid IMT among individuals with ASCVD may reflect the greater absolute values (i.e., minimized floor effect) in this subgroup.

## 5. Conclusions

Although the majority of fasting serum NEFAs were not associated with carotid IMT, estimated plaque thickness and FMD, serum non-esterified CLA in participants with ASCVD was positively associated with carotid IMT and non-esterified palmitoleic acid was positively associated with FMD in participants without ASCVD. This latter data suggests the potential value of CLA and non-esterified palmitoleic acid as biomarkers for arteriopathy.

## Figures and Tables

**Table 1:** Baseline (1996–1997) characteristics of participants in the Cardiovascular Health Study cohort.

Characteristics	Carotid IMT		FMD	
	No ASCVD ^a^ (*n* = 1314)	with ASCVD (*n* = 255)	No ASCVD (*n* = 1462)	with ASCVD (*n* = 301)
Age, years	77.3 ± 4.18	78.3 ± 4.35	77.4 ± 4.26	78.9 ± 4.66
Body mass index, kg/m^2^	26.8 ± 4.31	26.3 ± 3.98	26.9 ± 4.4	26.3 ± 4.05
Waist circumference, cm	96.1 ± 12.6	96.3 ± 11.4	96.5 ± 12.8	96.5 ± 11.6
Female, %	63.5	45.1	61.8	43.5
Black, %	14.0	14.5	14.0	15.3
CHS clinic, %				
California	31.1	26.1	29.6	30.6
Maryland	16.9	21.3	21.0	23.3
North Carolina	23.4	26.6	22.1	17.9
Pennsylvania	28.6	26.0	27.3	28.2
Educational attainment, %				
≥High school	54.6	49.2	52.1	46.3
Smoking status, %				
Never smoked	52.4	38.1	51.1	39.1
Former smoker	40.2	55.2	41.0	53.5
Current smoker	7.4	6.7	7.8	7.4
Alcoholic drinks/week, %				
0	53.7	51.2	54.7	53.8
1–7	34.3	36.6	33.1	34.8
>7	12.0	12.2	12.1	11.4
Hypertension, %	59.7	63.4	60.0	61.4
Diabetes, %	2.1	3.5	2.2	4.7
Prevalent AF, %	3.0	10.2	2.7	9.0
Prevalent CHF, %	0	30.6	0	33.2
Prevalent Stroke, %	0	27.8	0	27.6
Prevalent TIA, %	2.1	8.2	2.4	7.6
Prevalent PAD, %	0	13.3	0	12.0
Hypertension medication, %	47.4	73.7	48.4	72.4
Estrogen (females only), %	21.0	22.6	19.9	19.8
Fasting glucose, mg/dL	97.3 ± 12.8	99.3 ± 14.3	97.4 ± 12.7	100.2 ± 18.3
Albumin, g/dL	3.83 ± 0.29	3.82 ± 0.29	3.83 ± 0.29	3.82 ± 0.30
eGFR_cysc_^b^	74.2 ± 18.0	65.9 ± 18.8	74.0 ± 17.9	65.6 ± 18.4
C-Reactive Protein, mg/dL, log^2^	1.15 ± 1.57	1.42 ± 1.53	1.17 ± 1.56	1.49 ± 1.61
Carotid IMT, mm	2.13 ± 1.01	2.5 ± 1.17	-	-
FMD, % change	-	-	3.25 ± 2.05	3.00 ± 1.80

Values are presented as mean ± SD for continuous variables and percent for categorical variables. ^a^ Atherosclerotic cardiovascular disease (ASCVD) includes congestive heart failure, stroke, myocardial infarction and peripheral artery disease; ^b^ eGFR_cysc_, cystatin C for estimate glomerular filtration rate. AF, atrial fibrillation; Carotid IMT, carotid intima-media thickness; CHF, congestive heart failure; FMD, flow-mediated dilation; PAD, peripheral artery disease; TIA, transient ischemic attack.

**Table 2:** Selected associations of serum individual non-esterified fatty acid (NEFA) with carotid intima-media thickness (Carotid IMT) and flow-mediated dilation (FMD) in the Cardiovascular Health Study cohort, 1996–1997.

NEFAs, µmol/L per SD	No ASCVD ^a^		With ASCVD ^a^	
	Regression Coefficient (95% CI)	Adjusted *p*-Value ^b^	Regression Coefficient (95% CI)	Adjusted *p*-Value ^b^
**Carotid IMT**				
Sum of 18:1n-10–12*t* ^c^	3.61 (−5.09, 12.3)	1.00	39.4 (14.0, 64.7)	0.085
Elaidic acid, 18:1n-9*t*	−0.10 (−8.77, 8.58)	1.00	38.6 (14.1, 63.1)	0.077
Conjugated linoleic acid, 18:2*t*CLA	2.16 (−6.74, 11.1)	1.00	44.8 (19.2, 70.4)	0.025
**Flow-mediated dilation**				
Palmitoleic acid, 16:1n-7*c*	19.7 (8.34, 31.0)	0.024	4.91 (−17.4, 27.2)	0.667

Linear regression estimates are given per 1-SD increment in NEFA. CI, confidence interval. Regression model was adjusted for age, sex, race, field centers, education, smoking status, body mass index, physical activity, alcohol consumption, cystatin C for estimate glomerular filtration rate, serum albumin, diabetes, hypertension, use of anti-hypertensive, statin, and other lipid-lowering drugs. ^a^ Atherosclerotic cardiovascular disease (ASCVD) includes congestive heart failure, myocardial infarction, stroke and peripheral artery disease; ^b^ *p*-value was adjusted by Holm-Bonferroni procedure; ^c^ 18:1n10–12*t*, sum of 18:1n-10, n-11, and n-12 *trans* isomers.

## Data Availability

The data that support the findings of this study are available from the Cardiovascular Health Study (CHS) Coordinating Center upon approval of a signed data distribution agreement.

## Supplementary Materials

The following are available online at https://www.mdpi.com/article/10.3390/nu13093052/s1, Table S1: Median (interquartile range, IQR) for individual non-esterified fatty acids in each subgroup in the Cardiovascular Health Study participants at baseline (1996–1997), Table S2: Prospective association of fasting serum individual non-esterified fatty acid (NEFA) with carotid intima-media thickness (Carotid IMT) in the Cardiovascular Health Study cohort, 1996–1997, Table S3: Multivariable adjusted relative risk of fasting serum individual non-esterified fatty acid (NEFA) with estimated plaque thickness in the Cardiovascular Health Study cohort, 1996–1997, Table S4: Prospective association of fasting serum individual non-esterified fatty acid (NEFA) with flow-mediated dilation (FMD) in the Cardiovascular Health Study cohort, 1996–1997.
