# Supplementary material for: Serum Non-Esterified Fatty Acids, Carotid Artery Intima-Media Thickness and Flow-Mediated Dilation in Older Adults: The Cardiovascular Health Study (CHS)
Source: Nutrients. 2021 Aug 31;13(9):3052. doi: 10.3390/nu13093052 (PMC8465602; doi:10.3390/nu13093052)
Supplement: Supplementary file 1 [file nutrients-13-03052-s001.zip › nutrients-1317401-supplementary.pdf]

Serum non-esterified fatty acids, carotid artery intima-media thickness and flow-mediated dilation in older adults: the Cardiovascular Health Study (CHS)

Neil K. Huang,[a] Petra Bůžková,[b] Nirupa R. Matthan,[a] Luc Djoussé,[c] Jorge R. Kizer,[d] Kenneth J. Mukamal,[e] Joseph F. Polak,[f] Alice H. Lichtenstein,[a]

[a]Cardiovascular Nutrition Laboratory, Jean Mayer USDA Human Nutrition Research Center on Aging, Tufts University, Boston, MA; [b]Department of Biostatistics, University of Washington, Seattle, WA; [c]Division of Aging, Brigham and Women's Hospital, Harvard Medical School, Boston, MA; [d]Cardiology Section, San Francisco Veterans Affairs Health Care System, and Departments of Medicine, Epidemiology and Biostatistics, University of California San Francisco, San Francisco, CA; [e]Division of General Medicine, Beth Israel Deaconess Medical Center, Boston, MA; [f]Ultrasound Reading Center, Department of Radiology, Tufts Medical Center, Boston, MA

Corresponding author: Alice H. Lichtenstein, D.Sc.,

Jean Mayer USDA Human Nutrition Research Center on Aging

Tufts University

711 Washington Street

Boston, MA 02111

Phone: (617)556-3127

Supplemental Table S1. Median (interquartile range, IQR) for individual non-esterified fatty acids in each subgroup in the Cardiovascular Health Study participants at baseline (1996-1997)

NEFA, µmol/L	Carotid IMT		FMD	
	No ASCVD	With ASCVD	No ASCVD	With ASCVD
**SFA**	189.5 (77.3)	185.1 (86.6)	190.8 (77.7)	184.2 (84.7)
12:0	2.02 (1.65)	2.11 (1.59)	2.04 (1.66)	2.09 (1.65)
14:0	8.18 (4.84)	7.72 (4.28)	8.09 (4.82)	7.80 (4.30)
15:0	1.54 (0.65)	1.47 (0.61)	1.53 (0.65)	1.47 (0.59)
16:0	116.5 (53.2)	111.9 (55.3)	116.7 (54.0)	111.9 (54.4)
18:0	58.0 (20.5)	57.7 (23.1)	58.2 (20.9)	57.2 (22.0)
20:0	0.63 (0.31)	0.62 (0.34)	0.62 (0.30)	0.62 (0.35)
22:0	0.39 (0.14)	0.40 (0.16)	0.39 (0.14)	0.40 (0.16)
24:0	0.61 (0.24)	0.61 (0.24)	0.61 (0.25)	0.60 (0.24)
**MUFA**	170.0 (104)	157.2 (96.9)	171.2 (102.6)	157.2 (91.9)
14:1n-5	0.69 (0.70)	0.67 (0.62)	0.69 (0.70)	0.67 (0.59)
16:1n-9	1.85 (1.06)	1.70 (1.04)	1.84 (1.05)	1.73 (0.98)
16:1n-7	13.5 (12.3)	12.7 (11.5)	13.6 (12.0)	12.9 (11.0)
18:1n-9	141 (81.7)	132.3 (78.2)	141.1 (80.9)	132.9 (74.2)
18:1n-7	10.2 (6.95)	9.66 (6.58)	10.4 (6.77)	9.76 (6.36)
20:1n-9	0.93 (0.59)	0.88 (0.54)	0.94 (0.58)	0.89 (0.56)
22:1n-9	0.33 (0.20)	0.30 (0.19)	0.33 (0.21)	0.32 (0.19)
24:1n-9	0.32 (0.11)	0.33 (0.12)	0.32 (0.11)	0.33 (0.13)
**n-6 PUFA**	81.5 (47.0)	77.6 (45.1)	81.3 (46.3)	78.6 (44.0)
18:2n-6	73.1 (43.3)	69.1 (41.3)	73.0 (42.6)	69.7 (39.4)

18:3n-6	0.48 (0.38)	0.46 (0.38)	0.49 (0.36)	0.45 (0.36)
20:2n-6	0.80 (0.49)	0.80 (0.46)	0.81 (0.49)	0.80 (0.48)
20:3n-6	0.78 (0.56)	0.73 (0.56)	0.78 (0.53)	0.71 (0.56)
20:4n-6	4.69 (2.75)	4.51 (2.73)	4.67 (2.77)	4.48 (2.65)
22:4n-6	0.59 (0.42)	0.59 (0.48)	0.60 (0.43)	0.60 (0.45)
22:5n-6	0.34 (0.21)	0.31 (0.17)	0.34 (0.20)	0.31 (0.17)
**n-3 PUFA**	10.8 (5.70)	10.3 (6.04)	10.8 (5.56)	10.3 (6.06)
18:3n-3	5.18 (3.48)	5.04 (3.49)	5.18 (3.44)	4.92 (3.68)
18:4n-3	1.91 (1.23)	1.94 (1.21)	1.92 (1.25)	1.92 (1.20)
20:5n-3	0.29 (0.27)	0.27 (0.27)	0.29 (0.26)	0.27 (0.27)
22:5n-3	0.77 (0.51)	0.70 (0.50)	0.77 (0.49)	0.73 (0.51)
22:6n-3	2.10 (1.48)	1.92 (1.40)	2.07 (1.43)	1.92 (1.41)
***trans* fatty acid**	12.0 (6.96)	11.5 (7.31)	12.1 (7.00)	11.6 (7.14)
16:1n-9*t*	0.78 (0.56)	0.72 (0.59)	0.80 (0.55)	0.74 (0.57)
16:1n-7*t*	0.81 (0.45)	0.77 (0.47)	0.81 (0.44)	0.79 (0.46)
18:1n-10-12*t*[a]	0.63 (0.41)	0.59 (0.41)	0.63 (0.41)	0.60 (0.42)
18:1n-9*t*	5.98 (3.71)	5.73 (3.97)	6.06 (3.64)	5.81 (3.74)
18:1n-7*t*	2.51 (1.51)	2.31 (1.64)	2.54 (1.50)	2.30 (1.60)
18:2*t*[b]	0.18 (0.19)	0.18 (0.20)	0.18 (0.19)	0.17 (0.22)
18:2CLA	0.84 (0.86)	0.74 (0.69)	0.84 (0.88)	0.75 (0.80)

Values are median and interquartile range. Participants were classified as with (n=255 for carotid IMT, 301 for FMD) or without (n=1,314 for carotid IMT, 1462 for FMD) known atherosclerotic cardiovascular disease (ASCVD). Carotid IMT, carotid intima-media thickness; FMD, flow-mediated dilation; MUFA, monounsaturated fatty acid; PUFA, polyunsaturated fatty acid; SFA, saturated fatty acid. [a]18:1n10-12*t*, sum of 18:2n-10, n-11, and n-12 *trans* isomers; [b]18:2*t*, sum of all 18:2 *trans* isomers.

Supplemental Table S2. Prospective association of fasting serum individual non-esterified fatty acid (NEFA) with carotid intima-media thickness (Carotid IMT) in the Cardiovascular Health Study cohort, 1996-1997

	No ASCVD[a]		With ASCVD[a]	
**NEFA, umol/L per SD**	**Regression Coefficient (95% CI)**	**Adjusted *P*-value[b]**	**Regression Coefficient (95% CI)**	**Adjusted *P*-value[b]**
**SFA**				
12:0	2.78 (-5.46, 11.0)	1.000	4.5 (-18.0, 27.0)	1.000
14:0	-1.78 (-10.8, 7.28)	1.000	13.5 (-12.1, 39.0)	1.000
15:0	0.18 (-8.72, 9.07)	1.000	17.5 (-7.0, 41.9)	1.000
16:0	0.54 (-8.56, 9.64)	1.000	19.7 (-6.46, 45.8)	1.000
18:0	0.93 (-7.49, 9.34)	1.000	20.3 (-3.01, 43.5)	1.000
20:0	5.99 (-2.71, 14.7)	1.000	7.95 (-15.0, 30.9)	1.000
22:0	5.97 (-2.34, 14.3)	1.000	-5.39 (-28.6-17.8)	1.000
24:0	0.87 (-7.26, 9.00)	1.000	-7.06 (-29.9, 15.8)	1.000
**MUFA**				
14:1n-5	0.50 (-8.69, 9.70)	1.000	10.4 (-15.3, 36.1)	1.000
16:1n-9	1.23 (-8.03, 10.5)	1.000	20.3 (-6.97, 47.6)	1.000
16:1n-7	3.67 (-5.56, 12.9)	1.000	12.6 (-13.4, 38.7)	1.000
18:1n-9	1.41 (-7.67, 10.5)	1.000	25.0 (-1.38, 51.3)	1.000
18:1n-7	5.10 (-4.01, 14.2)	1.000	24.0 (-2.25, 50.2)	1.000
20:1n-9	3.80 (-4.76, 12.4)	1.000	22.1 (-2.21, 46.4)	1.000
22:1n-9	-4.27 (-12.6, 4.06)	1.000	-1.01 (-24.3, 22.3)	1.000
24:1n-9	4.82 (-3.42, 13.1)	1.000	-2.81 (-25.7, 20.1)	1.000
**n-6 PUFA**				

18:2n-6	-0.79 (-9.83, 8.24)	1.000	14.5 (-11.2, 40.2)	1.000
18:3n-6	-3.93 (-12.8, 4.95)	1.000	1.58 (-26.3, 29.5)	1.000
20:2n-6	2.92 (-5.93, 11.8)	1.000	23.6 (-0.31, 47.5)	1.000
20:3n-6	6.78 (-1.69, 15.2)	1.000	-2.18 (-26.3, 21.9)	1.000
20:4n-6	3.81 (-4.70, 12.3)	1.000	-13.9 (-38.5, 10.8)	1.000
22:4n-6	-2.42 (-11.2, 6.33)	1.000	15.3 (-9.26, 39.8)	1.000
22:5n-6	5.32 (-3.26, 13.9)	1.000	4.93 (-20.6, 30.5)	1.000
**n-3 PUFA**				
18:3n-3	-7.27 (-16.4, 1.86)	1.000	16.0 (-9.04, 41.1)	1.000
18:4n-3	2.15 (-6.50, 10.8)	1.000	4.46 (-20.5, 29.4)	1.000
20:5n-3	-1.69 (-10.1, 6.76)	1.000	3.18 (-20.5, 26.9)	1.000
22:5n-3	-0.61 (-9.35, 8.13)	1.000	13.5 (-11.1, 38.1)	1.000
22:6n-3	1.44 (-7.17, 10.1)	1.000	2.71 (-21.2, 26.6)	1.000
***trans* FA**				
16:1n-9T	4.46 (-4.67, 13.6)	1.000	34.8 (9.06, 60.6)	0.276
16:1n-7T	1.54 (-7.25, 10.3)	1.000	23.2 (-1.97, 48.4)	1.000
18:1n10-12T[c]	3.61 (-5.09, 12.3)	1.000	39.4 (14.0, 64.7)	0.085
18:1n-9T	-0.095 (-8.77, 8.58)	1.000	38.6 (14.1, 63.1)	0.077
18:1n-7T	0.164 (-8.41, 8.74)	1.000	30.5 (6.09, 55.0)	0.439
18:2T[d]	8.88 (0.449, 17.3)	1.000	11.4 (-11.5, 34.3)	1.000
**18:2CLA**	2.16 (-6.74, 11.1)	1.000	**44.8 (19.2, 70.4)**	**0.025**

Linear regression estimates are given per1-SD increment in NEFA. CI, confidence interval. Regression model was adjusted for age, sex, race, field centers, education, smoking status, body mass index, physical activity, alcohol consumption, cystatin C for estimate glomerular filtration

rate, serum albumin, diabetes, hypertension, use of anti-hypertensive, statin, and other lipid-lowering drugs. CLA, conjugated linoleic acid. [a]Atherosclerotic cardiovascular disease (ASCVD) includes congestive heart failure, myocardial infarction, stroke and peripheral artery disease; [b]*P*-value was adjusted by Holm-Bonferroni procedure; [c]18:1n10-12*t*, sum of 18:2n-10, n-11, and n-12 *trans* isomers; [d]18:2*t*, sum of all 18:2 *trans* isomers.

Supplemental Table S3. Multivariable adjusted relative risk of fasting serum individual non-esterified fatty acid (NEFA) with estimated plaque thickness in the Cardiovascular Health Study cohort, 1996-1997

NEFA, umol/L per SD	No ASCVD[a]	
	Relative risk (95% CI)	*P*-value
**SFA**		
12:0	0.99 (0.94, 1.04)	0.749
14:0	0.95 (0.81, 1.13)	0.570
15:0	1.07 (0.94, 1.21)	0.324
16:0	1.01 (0.85, 1.19)	0.921
18:0	0.97 (0.87, 1.09)	0.651
20:0	0.99 (0.92, 1.07)	0.770
22:0	1.01 (0.95, 1.07)	0.757
24:0	0.92 (0.83, 1.03)	0.169
**MUFA**		
14:1n-5	1.06 (0.92, 1.21)	0.446
16:1n-9	0.89 (0.77, 1.02)	0.086
16:1n-7	0.93 (0.77, 1.13)	0.450
18:1n-9	0.95 (0.73, 1.24)	0.713
18:1n-7	1.21 (0.98, 1.50)	0.078
20:1n-9	0.95 (0.83, 1.09)	0.447
22:1n-9	1.01 (0.96, 1.06)	0.781
24:1n-9	0.99 (0.95, 1.04)	0.792
**n-6 PUFA**		
18:2n-6	1.04 (0.87, 1.23)	0.670

18:3n-6	0.98 (0.91, 1.05)	0.498
20:2n-6	1.03 (0.95, 1.11)	0.482
20:3n-6	1.01 (0.92, 1.11)	0.833
20:4n-6	0.99 (0.91, 1.08)	0.878
22:4n-6	1.00 (0.94, 1.06)	0.962
22:5n-6	1.02 (0.94, 1.09)	0.674
**n-3 PUFA**		
18:3n-3	0.93 (0.83, 1.04)	0.212
18:4n-3	1.01 (0.96, 1.07)	0.663
20:5n-3	1.00 (0.91, 1.08)	0.905
22:5n-3	1.06 (0.94, 1.19)	0.347
22:6n-3	0.98 (0.89, 1.07)	0.642
***trans* FA**		
16:1n-9T	1.04 (0.91, 1.19)	0.530
16:1n-7T	1.00 (0.88, 1.13)	0.992
18:1n10-12T[b]	1.00 (0.86, 1.17)	0.956
18:1n-9T	1.01 (0.88, 1.15)	0.939
18:1n-7T	0.96 (0.85, 1.10)	0.563
18:2T[c]	1.04 (0.99, 1.09)	0.100
18:2CLA	0.95 (0.88, 1.01)	0.111

Poisson regression estimates are given per1-SD increment in NEFA. CI, confidence interval. Regression model was adjusted for age, sex, race, field centers, education, smoking status, body mass index, physical activity, alcohol consumption, cystatin C for estimate glomerular filtration rate, serum albumin, diabetes, hypertension, use of anti-hypertensive, statin, and other lipid-lowering drugs. CLA, conjugated linoleic acid. [a]Atherosclerotic cardiovascular disease

(ASCVD) includes congestive heart failure, myocardial infarction, stroke and peripheral artery disease; [b]18:1n10-12*t*, sum of 18:2n-10, n-11, and n-12 *trans* isomers; [c]18:2*t*, sum of all 18:2 *trans* isomers.

Supplemental Table S4. Prospective association of fasting serum individual non-esterified fatty acid (NEFA) with flow-mediated dilation (FMD) in the Cardiovascular Health Study cohort, 1996-1997

NEFA, umol/L Per SD	No ASCVD*		With ASCVD*	
	**Regression Coefficient (95% CI)**	**Adjusted *P*-value†**	**Regression Coefficient (95% CI)**	**Adjusted *P*-value†**
**SFA**				
12:0	4.40 (-5.80, 14.6)	1.000	-4.13 (-23.7, 15.5)	1.000
14:0	12.0 (0.84, 23.1)	1.000	0.23 (-21.6, 22.0)	1.000
15:0	9.88 (-1.04, 20.8)	1.000	10.6 (-10.4, 31.6)	1.000
16:0	10.7 (-0.52, 21.8)	1.000	4.97 (-17.2, 27.1)	1.000
18:0	0.11 (-10.3, 10.5)	1.000	16.2 (-3.83, 36.2)	1.000
20:0	-2.76 (-13.6, 8.12)	1.000	25.9 (6.34, 45.4)	0.350
22:0	-6.97 (-17.5, 3.56)	1.000	15.8 (-4.18, 35.7)	1.000
24:0	-3.11 (-13.2, 6.98)	1.000	-3.50 (-23.3, 16.3)	1.000
**MUFA**				
14:1n-5	17.1 (5.82, 28.4)	0.102	-2.65 (-24.8, 19.5)	1.000
16:1n-9	16.9 (5.53, 28.3)	0.121	3.84 (-19.2, 26.9)	1.000
**16:1n-7**	**19.7 (8.34, 31.0)**	**0.024**	4.91 (-17.4, 27.2)	1.000
18:1n-9	14.0 (2.82, 25.1)	0.439	7.95 (-14.2, 30.1)	1.000
18:1n-7	15.0 (3.81, 26.2)	0.279	5.01 (-17.3, 27.4)	1.000
20:1n-9	10.2 (-0.33, 20.8)	1.000	4.53 (-16.1, 25.1)	1.000
22:1n-9	9.72 (-0.66, 20.1)	1.000	5.74 (-14.3, 25.7)	1.000
24:1n-9	-2.04 (-12.3, 8.24)	1.000	-11.2 (-31.2, 8.84)	1.000
**n-6 PUFA**				

18:2n-6	11.9 (0.70, 23.0)	1.000	5.07 (-16.5, 26.6)	1.000
18:3n-6	5.15 (-5.88, 16.2)	1.000	9.48 (-13.7, 32.7)	1.000
20:2n-6	9.63 (-1.36, 20.6)	1.000	2.34 (-18.1, 22.8)	1.000
20:3n-6	2.74 (-7.74, 13.2)	1.000	3.65 (-17.0, 24.3)	1.000
20:4n-6	2.37 (-8.17, 12.9)	1.000	11.6 (-9.45, 32.6)	1.000
22:4n-6	3.80 (-6.97, 14.6)	1.000	-1.85 (-22.6, 18.8)	1.000
22:5n-6	11.0 (0.37, 21.7)	1.000	0.91 (-20.6, 22.4)	1.000
**n-3 PUFA**				
18:3n-3	9.00 (-2.25, 20.3)	1.000	-6.96 (-28.6, 14.6)	1.000
18:4n-3	7.82 (-2.96, 18.6)	1.000	4.41 (-17.0, 25.8)	1.000
20:5n-3	-0.047 (-10.5, 10.4)	1.000	8.00 (-12.6, 28.6)	1.000
22:5n-3	4.75 (-6.02, 15.5)	1.000	6.59 (-14.8, 28.0)	1.000
22:6n-3	1.76 (-8.88, 12.4)	1.000	-0.70 (-21.5, 20.1)	1.000
***trans* FA**				
16:1n-9T	12.0 (0.71, 23.3)	1.000	-0.52 (-22.9, 21.8)	1.000
16:1n-7T	3.93 (-6.93, 14.8)	1.000	3.74 (-17.6, 25.1)	1.000
18:1n10-12T‡	1.35 (-9.49, 12.2)	1.000	-0.15 (-21.7, 21.4)	1.000
18:1n-9T	-3.55 (-14.3, 7.24)	1.000	2.85 (-18.0, 23.7)	1.000
18:1n-7T	-7.76 (-18.4, 2.87)	1.000	-3.77 (-24.6, 17.0)	1.000
18:2T§	8.46 (-1.93, 18.8)	1.000	0.87 (-19.1, 20.8)	1.000
18:2CLA	11.2 (0.17, 22.2)	1.000	5.00 (-17.4, 27.4)	1.000

Linear regression estimates are given per1-SD increment in NEFA. CI, confidence interval. Regression model was adjusted for age, sex, race, field centers, education, smoking status, body mass index, physical activity, alcohol consumption, cystatin C for estimate glomerular filtration

rate, serum albumin, diabetes, hypertension, use of anti-hypertensive, statin, and other lipid-lowering drugs. CLA, conjugated linoleic acid. [*]Atherosclerotic cardiovascular disease (ASCVD) includes congestive heart failure, myocardial infarction, stroke, and peripheral artery disease; [†]*P*-value was adjusted by Holm-Bonferroni procedure; [‡]18:1n10-12*t*, sum of 18:2n-10, n-11, and n-12 *trans* isomers; [§]18:2*t*, sum of all 18:2 *trans* isomers.
